# Physico-Chemical and Ecotoxicological Evaluation of Marine Sediments Contamination: A Case Study of Rovinj Coastal Area, NE Adriatic Sea, Croatia

**DOI:** 10.3390/toxics10080478

**Published:** 2022-08-16

**Authors:** Jadranka Pelikan, Nina Majnarić, Maja Maurić Maljković, Kristina Pikelj, Bojan Hamer

**Affiliations:** 1 Faculty of Science, Department of Geology, University of Zagreb, Horvatovac 102a, 10000 Zagreb, Croatia; 2 Laboratory for Marine Nanotechnology and Biotechnology, Center for Marine Research, Ruđer Bošković Institute, Giordano Paliaga 5, 52210 Rovinj, Croatia; 3 Faculty of Veterinary Medicine, University of Zagreb, Heinzelova 55, 10000 Zagreb, Croatia

**Keywords:** Adriatic Sea, marine sediments, heavy metals, PAHs, PCBs, phytotoxicity, probability of toxic effects, French marine sediment quality guidelines

## Abstract

Comprehensive spatial and temporal data on sediment quality in the Adriatic Sea are lacking. Therefore, prior to planned anthropogenic interventions in the local marine environment, such as deepening of the Rovinj harbour, the results of physicochemical and ecotoxicological analyses of five local coastal sediments were compared with regional averages and SQGs of neighbouring countries. Analyses of sediment grain size, content of metals and heavy metals, PAHs and PCBs were performed according to standard protocols. Sediment quality was classified according to French legislation (N1 and N2 level) and sediment guidelines. The phytotoxicity of the eluates was studied by flax seed germination tests. The logistic regression models P_max_ and P_avg_ were used to estimate the probability of toxic effects. Except for the open sea (S5), all other sediments had concentrations slightly higher than the N1 for some metals (Cu, Ni, Hg, Cr) or ΣPAHs, while the Rovinj harbour (S1) reached the N2 value for mercury. The phytotoxicity assay with sediment eluates showed inhibition of germination, root length and root biomass production, with an average phytotoxicity index (PI) ranging from 6.06% to 42.00%. Significant correlations of P_avg_ and P_max_ values with phytotoxicity and other specific parameters were found. In general, according to the applied SQGs, there are no potential ecological impacts on the marine environment at any of the investigated sites, with the exception of site S1.

## 1. Introduction

Sediments are considered a suitable medium for studying pollution of aquatic environments because they represent a sink for a variety of pollutants over a period of time. In addition, dredging of seaports is currently very important to maintain marine depths in local and commercial harbours. Dredged sediments are often heavily contaminated, leading to waste disposal problems. Mediterranean coastal sediments are particularly heavily contaminated with metals and organic compounds such as organotin compounds, polycyclic aromatic hydrocarbons (PAHs) and polychlorinated biphenyls (PCBs) [1]. Contaminated sediments can pose a serious risk to aquatic organisms and ecosystems, and there has been increasing interest in developing new science-based assessment criteria for setting priorities and making management decisions [2,3].

The Adriatic Sea is a semi-enclosed basin connected to the eastern Mediterranean by the Otranto Strait [4]. It is usually divided into the northern, central and southern Adriatic, with the northern part being the shallowest. The total length of the Croatian Adriatic coast is 6278 km (mainland 1880 km; islands 4398 km). Data on sediment quality in the eastern Adriatic in terms of the content of metals and heavy metals, PAHs and PCBs are still quite scarce. However, the existing data are urgently needed to define limits for pollutants in sediments for different uses of marine resources: e.g., bays, beaches, villages, harbours, mariculture sites, marinas, marina service areas and others [5]. Until 2022, there was a lack of national Croatian regulations for assessing the quality of marine sediments with limit values and sediment categories [6,7,8,9,10].

Sediment quality guidelines (SQGs) are an important tool for assessing pollution of marine and estuarine sediments. Although such guidelines are not definitive indicators of toxicity, they can be highly predictive and useful for identifying areas of potential adverse biological impact. Despite the fact that the weight-of-evidence (WOE) approach was developed in combination with multiple lines of evidence (LOEs) for sediment characterization, some regulatory frameworks still rely on chemical characterization relative to SQGs as stand-alone decision criteria [11,12,13]. Different approaches for deriving sediment quality criteria are widely used in Europe, the USA, Canada and Australia [14]. For example, the sediment quality guidelines proposed by Long et al. [15] and the US EPA [16] based on biological toxicity tests of the benthic environment [17] were developed as a composite indicator divided into three levels with effect range low (ERL) and effect range median (ERM) values. In environmental toxicology, ERL and ERM are measures of toxicity in marine sediments. They are used by public agencies in the United States in formulating guidelines for assessing toxicity hazards, particularly from trace metals or organic contaminants [16].

The ERL and ERM measures are expressed as specific chemical concentrations of a toxic substance in sediment. The ERL indicates the concentration below which toxic effects are rarely observed or predicted; the ERM indicates the concentration above which effects are generally or always observed [18]. They are not regulatory criteria and should not be used as such. The US EPA uses ERL and ERM values as a type of “benchmark” for sediments. They define a benchmark as a concentration that, if exceeded, has the potential to cause harm or significant risk to humans or animals in the environment.

The neighbouring country of Croatia, Italy, uses integrated WOE criteria [19]. Such multidisciplinary WOE approaches include integration of different lines of evidence (LOEs): 1. chemical characterization of sediments; 2. bioavailability of chemicals; 3. sublethal effects—biomarkers; 4. ecotoxicological bioassays; 5. analysis of benthic communities after classification into WOE risk classes (absent, slight, moderate, major, and severe) [13,20,21,22,23]. Considering the triad of sediment quality, a consensus on full chemical and ecotoxicological characterization and WOE risk assessment of (contaminated) sediments should balance availability, financial costs, scientific benefits and cost advantages [24,25].

Even at the EU level, regulations and types of sediment analysis vary from country to country, and the European Commission does not require Member States to adopt a particular approach [26]. In general, the chemical criteria approach should be supported by bioassays, bioaccumulation tests and ecological analyses to define an integrated assessment of contaminated sediments and better evaluate toxic effects on benthic organisms and impacts on aquatic ecosystems. Such assessment frameworks should be based on broad consensus and be flexible and adaptable as new data become available.

It is possible that the full implementation of the Water Framework Directive (WFD (2000/60/EC) and the Marine Strategy Framework Directive (MSFD 2008/56/EC), as well as the national setting of values for Good Environmental Status (GES) will accelerate the national coastal sediments screening and establishment of sediment quality guidelines. In general, the strategy for developing guidance values/threshold values is based on a statistical evaluation of pollutant concentrations, measured during multiannual campaigns. Examining the distribution of the results allows for determining the “background noise” value, i.e., the natural content with no discernible anthropological contribution. A common approach for conducting a screening sediment risk assessment is to compare available chemical data on sediments with SQGs.

French regulations and contamination guidance values were used to assess Croatian sediment quality, since both countries are Mediterranean countries with intensive aquaculture, tourism and marine traffic activities. The French regulations for the management of contaminated sediments are based on two levels (N1 and N2 threshold values) of contaminant concentrations in the bulk sediment (JORF No. 184, 10-08-2000) [1,27,28]. The French SQGs for the management of contaminated dredged sediments include several chemicals: metals (As, Cd, Cu, Ni, Pb, Zn, Hg and Cr), ΣPAHs and ΣPCBs analyses [1,27,28]. Dredged sediment disposal is allowed offshore if the concentrations of metal contaminants is lower than level 2 (N2). Disposal may be prohibited if concentrations are higher than N2. Dredged sediments that are considered highly contaminated (>N1) may not be discharged to the sea and must be treated and deposited at a terrestrial site before storage, where they are considered waste. The ecotoxicity of sediments must be then evaluated prior to disposal or further use. However, the legislation does not provide ecotoxicological tests to assess the impact of sediments on the terrestrial environments. Some authors [28] propose a framework for ecological risk assessment of dredged material that includes two steps: a simplified risk assessment based on contaminant concentrations and a risk assessment based on laboratory toxicity tests, such as phytotoxicity tests or bioassays with aquatic organisms (bacteria, algae). Several tests are widely used to assess sediment toxicity, such as the Microtox solid phase test. The framework for ecological risk assessment of dredged material includes knowledge of contaminant concentrations associated with laboratory toxicity tests [28].

The aim of this study was to analyse physico-chemical properties and perform a simply ecotoxicological assessment (phytotoxicity) of marine sediments along the coast of Rovinj before the planned deepening of the local harbour. Sediment samples from the Rovinj harbour as well as from neighbouring Lim Bay and the open sea are used for comparison. The assessment is based on the French legislation for sediment quality criteria in the context determining the level of contamination, with a contribution to the development of national criteria for sediment quality (indicative values/threshold levels) and the assessment of good ecological status (GES) for sediments [6,7].

## 2. Materials and Methods

### 2.1. Sediment Sampling

**Figure 1:**
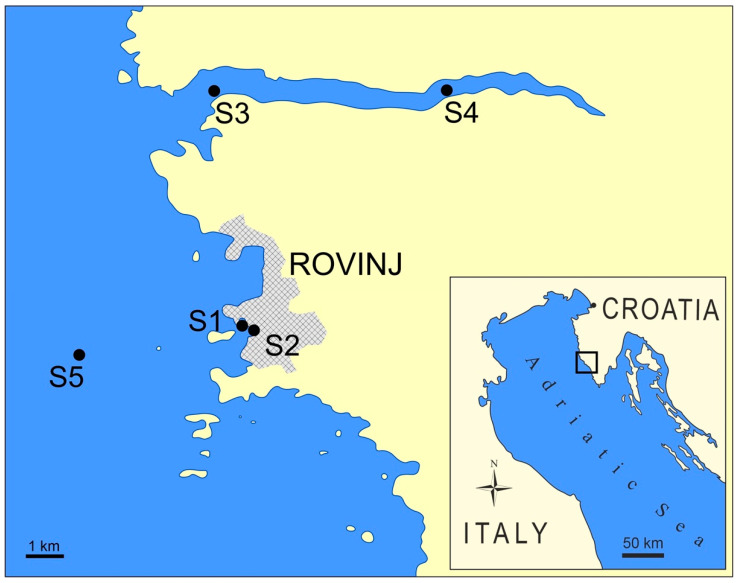
Location of the sampling sites in NE Adriatic Sea: S1—Local harbour, S2—Shipyard, S3—Lim Bay out, S4—Lim Bay middle and S5—Open sea (3 NM in front of Rovinj city).

Five sampling sites (Figure 1) were investigated in the eastern Adriatic Sea near the city of Rovinj. The sampling sites differ according to the anthropogenic impacts such as traffic, industrial and urban runoff waste of Rovinj City with 12,000 inhabitants: Local harbour (S1–N 45°04′46.4, E 13°38′03.5) and Shipyard (S2–N 45°04′37.3, E 13°37′59.6); Lim Bay out (S3–N 45°08′02.0, E 13°37′31.4) and Lim Bay middle (S4–N 45°07′49.4, E 13°40′15.4) under the traffic and mariculture influence; Open sea 3 NM from Rovinj (S5–N 45°05′16.6, E 13°32′51.4) was used as the reference site (Figure 1). Surface sediment samples were collected in August 2011 from selected sites at depths ranging from 6 to 30 m. Sediments were sampled using a Van Veen grab.

### 2.2. Sediment Grain Size Analysis

The top 5 cm of undisturbed sediment from each sediment sample was carefully subsampled through the top window of the grab. Prior to further subsampling, the collected 5 cm surface sediment was carefully homogenized with a firm plastic spoon. The homogenized sediment samples were stored at +4 °C immediately after sampling and then stored at −80 °C in the laboratory until analysis. Prior to grain size analysis, sediment samples were defrosted and weighed. Their dry weight was calculated as the weight loss after drying at 70 °C for 24 h. Wet sieving method with Retsch sieves was used to separate coarse-grained (>0.063 mm) and fine-grained fractions (<0.063 mm). The coarse-grained fractions were dried and further dry-sieved using a set of 7 sieves with the following mesh sizes: 4 mm, 2 mm, 1 mm, 0.5 mm, 0.25 mm, 0.125 mm and 0.063 mm. The mud fraction was not further separated into silt and clay. The sediment retained on each sieve was dried and weighed, while the weight of the fine-grained (mud) fraction was calculated using the initial dry weight. The GRADISTAT statistical package [29] was used for the basic granulometric description: sediments were classified according to the Folk scheme [30], while the descriptive granulometric statistics were calculated using the formulas proposed by [31].

### 2.3. Analyses of Contaminant Concentrations

#### 2.3.1. PAHs

PAHs were analysed by the Primorsko-goranska County Teaching Institute of Public Health and quantified as described by [32]. Previously dried sediment (10 g) was extracted with 100 mL of spectrograde cyclohexane (Merck, Darmstadt, Germany) in an ultrasonic bath for 45 min. After filtration, the extract was evaporated to dryness using a rotary evaporator. The residue was again dissolved in 1 mL of cyclohexane and the PAH fraction was separated on a column with 1 g of silica-gel (Kemika, Zagreb, Croatia) and with some Al_2_O_3_ on top. The column was eluted with 25 mL of cyclohexane and the eluate was evaporated to near dryness on a rotary evaporator. The remaining PAH fraction was dissolved in 1 mL of distilled methanol and analysed by HPLC.

Thirteen PAHs, Fluorene (1.00 μg/kg detection limit), Phenanthrene (1.00 μg/kg), Anthracene (1.00 μg/kg), Fluoranthene (2.00 μg/kg), Pyrene (1.00 μg/kg), Chrysen (1.00 μg/kg), Benzo(a)anthracene (1.00 μg/kg), Benzo(b)fluoranthene (1.00 μg/kg), Benzo(k)fluoranthene (1.00 μg/kg), Benzo(a)pyrene (1.00 μg/kg), Dibenzo(a,h)anthracene (1.00 μg/kg), Benzo(g,h,i)perylene (1.00 μg/kg) and Indeno(1,2,3-c,d)pyrene (1.00 μg/kg), were identified and quantified using Supelco internal standards using an HPLC module system HP 1050 (Hewlett Packard, Palo Alto, CA, USA). This system consisted of a quaternary pump with a rheodine valve injector (HP 7125) and a 20 µL sampling loop with a variable wavelength UV detector and integrator (HP 3396) as previously described [33]. PAHs were separated on a reversed phase cartridge column HP Li Chrospher 100 RP-18 (51 m, 25 × 4 mm).

#### 2.3.2. PCBs

Approximately 20 g of the wet sediment was homogenised with anhydrous NaSO_4_ and extracted with n-hexane according to [33]. Extracts were analysed using GC/MS –QP2010 (Shimadzu Corp., Kyoto, Japan) equipped with an AOC-5000 autoinjector (CTC Analytics AG, Twingen, Switzerland). PCBs were analysed by the Teaching Institute of Public Health, Primorsko-goranska County, Rijeka, and quantified as Aroclor 1260.

#### 2.3.3. Metals’ Concentrations

Metals were analysed and quantified by the Primorsko-goranska County Teaching Institute of Public Health, as previously described by [33]. Sediment samples were dried overnight in an oven at 105 °C. The samples (1 g, <2 mm grain size fraction) were then heated with HNO_3_ in a microwave oven (PerkinElmer/Anton Paar Multiwave 3000). Metal content was determined using a Perkin Elmer (Waltham, MA, USA) 4110 ZL (Pb, Hg, and Cd) and a Perkin Elmer Analyser 200 (Cu and Zn). Pb and Cd were determined by the graphite furnace atomic absorption spectroscopy (GFAAS) method, while Hg was determined by the flow injection mercury system (FIMS) method. Cu, Zn and the other metals were determined by direct flame atomization.

#### 2.3.4. Evaluation of Sediment Contamination Based on French Sediment Quality Guidelines

To evaluate the contamination of the studied sediments, the French sediment quality guidelines (SQGs) were used with two guide levels (N1 and N2) for the concentrations of selected metals (As, Cd, Cu, Ni, Pb, Zn, Hg and Cr), ΣPAHs, and ΣPCBs in the total sediment [1,27]. Total PAHs are defined by the sum of 13 PAHs (Fluorene, Phenanthrene, Anthracene, Fluoranthene, Pyrene, Benzo(a)anthracene, Chrysene, Benzo(b)fluoranthene, Benzo(k)fluoranthene, Benzo(a)pyrene, Dibenzo(ah)anthracene, Benzo(ghi)perylene and Indeno(1,2,3,c,d) pyrene). The total PCBs are expressed as Aroclor 1260.

The ∑Q_N1_ and Q_PECm_ risk quotients were used as appropriate indicators of potential ecological effects on the aquatic environment under study. ∑Q_N1_ represents the sum of all individual ratios between the contaminant concentration and the French N1 legal level (PEC value). The general sediment risk quotient Q_PECm_ represents the averaged ∑Q_N1_ value for a given sediment divided by the number of all pollutants studied, taken from the French guidelines for the management of marine dredged sediment [27,28], as presented in Equation (1).

Q_PECm_ = (Σ^n^_i=1_ (C_i/_PEC_i_))/n (1)

where C_i_ is the measured contaminant concentration, PECi is the predicted effect concentration, and n is the number of measured contaminants (n = 10). Thus, the risk quotient (Q_PECm_) is less than 1 when the contaminant concentration is less than the N1 level, and greater than 1 when the contaminant concentration exceeds N1.

#### 2.3.5. Probability of Toxic Effects

To estimate the probability of a toxic effect, logistic regression models were applied to sediment contaminant concentration data, as proposed by Field et al. [34]. The authors describe individual logistic regression models for 37 chemicals of potential concern in contaminated sediments that link sediment chemistry to the probability of toxic effects in standard 10-d survival tests for the marine benthic amphipods *Ampelisca abdita* and *Rhepoxynius abronius*.

Equation (2) shows the calculation of the probability of toxic effects:

P = [exp (B_0_ + B_i_ (x))]/[1 + exp (B_0_ + B_i_ (x))] (2)

where P is the probability of observing a toxic effect, B_0_ is the intercept parameter, B_i_ is the slope parameter and x is the chemical concentration or log chemical concentration.

The logistic regression models for individual chemicals were combined to obtain a single probability of toxic effect using two approaches: (1) the maximum probability model (P_max_), which was derived from the single chemical model with the highest probability for a sample, and (2) the average probability model (P_avg_), which was derived from the arithmetic mean of the probabilities from models for all chemicals measured for a sample. The relationship between the maximum (or mean) toxicity probability of each chemical model and the proportion of toxic samples was described by a binomial least-squares regression model of the interval data. The binomial models were used to estimate the probability of toxicity for individual samples.

#### 2.3.6. Phytotoxicity Assay

For the phytotoxicity test, 4 g of dried sediment was eluted for 24 h with ultrapure water (1:10, sediment/water) according to [35]. The pH values of the sediment eluates ranged from 8.00 to 8.40. The germination test was performed with seeds of flax *Linum usitatissimum* at 25 °C for a test period of 72 h [1]. Flax seeds were purchased commercially (Bio Zone, Croatia), and sorted by size and appearance. For each replicate (3) of sediment sample, three sheets of filter papers were placed in 3 Petri dishes (100 × 15 mm). Three groups of thirty seeds were placed in each Petri dish (at least 0.5 cm from the edge of the filters) and 5 mL of eluates (corresponding to 100 g/L of sediment d.w.) or ultrapure water was added to filter papers for sediment samples and controls, respectively. The covered Petri dishes were placed in a humidified thermostat (HERA Cell 150, Heraeus) in the dark. After 24 h, the percentage of seed germination was determined. After 72 h, root biomass production was measured, with biomass of roots and seeds weighed separately, and finally, the sum of root elongation for each replicate of sediment samples was measured with a digital ruler for each seed root length.

The percentage of seed germination (SG), root growth measured as biomass production (BP), and root elongation inhibition (RL) compared to the test control (deH_2_O) was calculated as shown in Equation (3):

SG/BP/RL = (Ct − S)/Ct × 100 (3)

where Ct is the mean of seed germination, biomass, or total root elongation in the controls; and S is the mean of seed germination, biomass, or root elongation in the samples.

The total phytotoxicity index (PI) was calculated as the average of all three measured parameters: seed germination (SG), biomass production (BP) and root elongation inhibition (RL) (Table S3).

### 2.4. Statistical Analysis

Pearson product moment correlation coefficients, Kruskall-Wallis ANOVA (n = 3 per site) and multiple comparisons of mean ranks (with a Bonferroni adjustment) were calculated using Statistica v.14 TIBCO Software Inc.

## 3. Results and Discussion

### 3.1. Sediment Grain Size

Sediment samples S1 and S5 are the coarsest sediments from the analysed set with sand as the dominant fraction (>67% and >72%, respectively). Both, S1 and S5 are classified as very poorly sorted gravelly muddy sands (Table S1), with mean sizes of 167 and 175 µm, respectively (Table S1). Sample S2 is also dominated by sand (>54%); however, it contains a higher percentage of mud (~45%) compared to S1 and S5. Sample S2 is therefore classified as poorly sorted slightly gravelly muddy sand with a mean size of 72 µm. Sediment samples S1, S2 and S5 are typical eastern Adriatic coarse-grained sediments in which sand fraction dominates [36,37,38,39,40]. Biogenous detritus was dominant in the main sandy fractions, and according to various authors, most of these sediments consist of biogenous carbonate sand-sized particles mixed with a smaller amount of siliciclastic material, mostly in mud size [36,37,38,39,40].

Both samples from Lim Bay (S3 and S4) are classified as mud. Sample S3 is well sorted sandy gravelly mud and sample S4 is very well sorted slightly gravelly mud, while both their mean sizes are ~45 µm (Table S1). It must be understood, however, that no detailed separation of silt and clay was made for this study. Therefore, it is likely that the actual mean size may be even lower. The fine-grained nature of the Lim Bay sediments has already been described by [41,42]. In agreement with the carbonaceous character of the sand fraction along the eastern Adriatic and its low proportion in samples S3 and S4, it is expected that the Lim Bay sediment contains more siliciclastic material compared to samples S1, S2 and S5. Both the mineral composition and the detailed analysis of the mud fraction should be carried out in the future work.

### 3.2. PAHs

The results of this study (Table 1) indicate a relatively low concentration of PAHs in marine sediments collected at sites S4—Lim Bay middle (28.2 µg/kg d.w.) and S5—Open sea 3NM off Rovinj (103.0 µg/kg). The highest PAH concentrations were found at the sampling sites located in the local harbour–S1 (10,609.2 µg/kg) and S2—Shipyard (9867.1 µg/kg). In addition to the traffic and impact of the local shipyard, the sites were closely related and connected by the exchange of water masses. Interestingly, site S3 at the beginning of Lim Bay, which had previously been used as a reference site (Projekt Jadran 1998–2010), also had relatively high concentrations of PAHs (4555.9 µg/kg) in surface sediments [43]. This is probably due to the intensive traffic of small motorboats entering Lim Bay for sightseeing and aquaculture purposes. Some studies have shown that PAHs have a higher affinity for the fine-grained sediments in various environments, inside and outside of the Adriatic Sea [44,45]. However, the results of this study showed that the highest PAH concentrations were found in sediment samples of different textures (e.g., S1 and S3; Table S1). These results suggest that grain size does not play the main role in the distribution of PAHs in studied sediment. Instead, exposure to the marine traffic and associated activities appear to have the greatest influence on PAH concentrations, suggesting that the relationship between sediment and PAHs is quite complex and site-specific.

PAHs containing 4 to 6-ring hydrocarbons are generally pyrogenic in origin and are formed during the combustion of fossil fuels and recent organic material. The distribution patterns of PAHs (3 to 6-rings) shown in Table 1 and Figure S1 indicate that 4- and 5-ring PAHs dominate (70% at all sampling sites), consistent with pyrogenic origin. The enrichment of 4-ring PAHs, particularly fluoranthene and pyrene, indicates greater combustion of diesel fuel.

The PAH ratios can also be used to identify sources of contamination [46]. The ratios of Phe/Ant and Flt/Pyr have long been used to distinguish pyrogenic sources from petrogenic ones [47]. Ratios of Phe/Ant > 15 and Flt/Pyr < 1 indicate a dominance of petrogenic sources [48,49]. In addition, ratios of Ant/(Ant+Phe) < 0.1 and Flt/(Flt+Pyr) < 0.4 indicate petrogenic sources and ratios of Ant/(Ant+Phe) > 0.1 and Flt/(Flt+Pyr) ≥ 0.4–0.5 indicate pyrogenic sources [50]. A higher Flt/(Flt+Pyr) ratio > 0.5 has been used for grass, wood and coal combustion sources [51,52].

For all samples, it is not surprising that PAHs of pyrolytic origin are prevalent in the region due to the increased traffic of small and large motorboats. This finding is confirmed by a large body of published data indicating that pyrolytic sources dominate PAH contamination of marine sediments of the Mediterranean basin [32,43,53,54,55,56].

Some studies such as Bihari et al. [43,55] reported total concentrations of PAHs in marine sediments in the Rovinj and Rijeka area, ranging from 213.0 to 695.0 µg/kg d.w., while other studies such as Traven et al. [57,58] reported that the total concentration of PAHs in local sediment samples collected from the Kvarner Bay ranged from 113.8 µg/kg d.w. for a recreational area to 11,479.0 µg/kg d.w. for an industrial site. The author of [46] reported that in recent years the situation according to PAH levels in the Gulf of Trieste (Slovenia) falls within the concentration from 100 to 1000 ng/g (moderately polluted areas) with a reducing trend. Comparing the total PAH values (µg/kg d.w.) from [43] and the PAH values from this study at overlapping sites 10 years later, the reference site and the most polluted site (S4–35.76 ⇒ 28.2 µg/kg and S1–13,681.0 ⇒ 10,609.2 µg/kg, respectively), it can be concluded that PAH concentrations are generally slightly decreasing in the study area. However, it is not possible to conclude about decreasing trend for PAH contamination, because the values at the studied site S3 are surprising during the 10 year period: S3–31.98 ⇒ 4555.9 µg/kg [43,59]. It is likely that the fate of PAHs in marine ecosystems is determined by both biotic and abiotic parameters [60].

PAHs are toxic compounds and can have harmful biological effects. With the goal of ecological risk assessment, specific numeric sediment quality guidelines have been developed to evaluate the adverse biological effects of various contaminants. The SQGs for PAHs were derived from the results of numerous laboratory and field studies and various toxicity tests for different aquatic organisms [18,61]. The lower effect range (ERL) and the median effect range (ERM) have been used to evaluate potential adverse toxicological effects [18]. These ranges are used to define concentrations of contaminants that are rarely (<ERL), occasionally (≥ERL and <ERM) or frequently (≥ERM) associated with adverse biological effects. In this study, total concentrations of PAHs and individual concentrations of PAHs were compared with ERL and ERM values (Table 1). The results showed that the total concentrations of PAHs at S1, S2 and S3 sites were above the ERL and significantly lower than the ERM values. This means that the PAH concentrations in the sediments of the studied area at sites S1, S2 and S3 are in a range where harmful toxicological effects are expected.

### 3.3. Concentration of Metals

Comparing obtained results (Table 2) from the studied sites in Rovinj with the trace metal concentrations in surface sediments at most stations (National Monitoring Programme Projekt Jadran results) along the eastern Adriatic coast, it can be concluded with some exceptions that the Croatian coastal area is not significantly contaminated with trace metals [62]. The concentrations found in the sediments analyzed for this study correspond to the natural metal content of the Adriatic [63,64,65,66,67], and are within the range of values characteristic for low to moderately polluted areas of the Mediterranean [68,69,70,71]. Furthermore, the results of the Projekt Jadran have shown that sediment contamination is limited to very narrow coastal areas near urban pollution sources of pollution, so called “hot spots”, such as mercury (Hg) in the Kaštela Bay at the Inavinil station and lead (Pb) at the Vranjic station [62].

Elevated levels of some metals (Cu, Hg) were found near the city centre and ports (e.g., at study site S1; Table 2), due to high traffic intensity and the impact of certain pollution sources, such as industrial and municipal wastewater, harbour water, etc. [72]. Ni and Cr are elevated at the central Lim Bay (S4), probably due to traffic and sea currents carrying local industrial and municipal waste waters.

There are several studies on the impact of local industry on marine quality, e.g., INA refinery Rijeka–Kvarner Bay [55,57,58], with a spatial and temporal database of heavy metal contamination in sediments [5,73], including selected heavy metal data in sediments and mussel *Mytilus galloprovincialis* of the monitored hot spots [62]. For the purpose of analysing metal contamination of sediments, the authors [74] divided the sites into seven categories: 1—bays, 2—beaches, 3—villages, 4—ports, 5—marinas-pier areas, 6—marina service areas and 7—others (sea mud, river tributaries etc.). The concentration values of Category 1 sediment samples can be used in defining “normal” or ‘‘natural” background values for concentrations of chemical elements in coastal sediments. Based on these values, standards for dredged sediment disposal and intervention values could be derived. Furthermore, [74] provides descriptive statistics for the elements Cu, Zn, As and Pb for six categories of coastal sediment samples. Concentration values for all four elements show the same trend: they increase from bays and beaches to villages, ports, marina-piers and marina-service areas. In fact, the high concentrations measured at pollution sources decrease rapidly along the transport pathways of suspended matter [5]. Concentrations typically decrease by several orders of magnitude as little as 100 m from the pollution source. The full implementation of wastewater collection and treatment in the city of Rovinj in 2022 is expected to significantly reduce the input of pollution into the local marine environment.

Based on the granulometric composition of the sediments (predominant mud fraction) and the associated mineral composition (more clay minerals) [42], the fine-grained sediments from Lim Bay (S3 and S4) are expected to contain a significantly higher concentration of trace minerals. Compared to the sample from the open sea (S5), this trend is visible for all trace metals measured in Lim Bay (Table 2). Nevertheless, the coarse-grained sediment from the Rovinj harbour showed a considerable load of trace metals (Table 2), indicating a strong local influence of harbour activities, notwithstanding the low percentage of fine-grained particles.

### 3.4. PCBs

The PCB concentrations of <0.01–0.278 mg/kg d.w. measured in surface sediments in this study (Table 2) were lower than those previously found in the northern and central Adriatic: 0.9–14.7 mg/kg d.w. [75] or 3–80 mg/kg d.w. [76] and in the eastern Adriatic: <0.5–29.4 mg/kg d.w. [77], reflecting restrictions on the use and production of these compounds. This is consistent with studies showing a significant decrease in PCB concentrations in various environmental compartments of the Mediterranean and Adriatic Seas over the last two decades [78,79,80].

Regarding the relationship between grain size and PCBs, several studies have shown that PCBs preferentially accumulate in the fine-grained fraction of the sediment, similar to other pollutants (e.g., heavy metals, PAHs) and organic matter (OM) [81,82]. Therefore, one would expect a higher ΣPCBs concentration in Lim Bay sediments (S3 and S4; Table 2); however, the local effects of harbour and shipyard activities resulted in an order of magnitude higher PCB concentration in sediment samples S1 and S5. As expected, control site sample S5 contained the lowest concentration of PCBs (Table 2).

In general, total PCB concentrations detected in the southern Adriatic Sea were comparable to those detected in deep sediments (>620 m) of the eastern Mediterranean [83] and in coastal waters of Spain [84,85]. Total PCB concentrations in sediment cores collected near the Po delta were similar to those found in the northern Adriatic Sea [75,86], but lower than in more industrialized and urbanized areas, such as the Mar Piccolo of Taranto [56], the East China Sea [87,88], the Baltic Sea [89] and the Bizerte Lagoon in Tunisia [90].

PCBs are synthetic compounds that have been produced since the 1930s. In the Adriatic Sea, whose water renewal period is less than 10 years, there may have been an accumulation of residues of chlorinated compounds that were discharged through the atmosphere. Moreover, additional amounts of these pollutants have been added to the sea from various local sources (mainly from sewage and industrial effluent disposal, port activities and agricultural drainage) located along the coast, including semi-enclosed bays [91].

### 3.5. Sediment Quality Evaluation

Chemical analyses of total sediments and French guidance-threshold levels (N1 and N2) are shown in Table 2. Several contaminants were present at concentrations higher than the N1 level (Cu, Ni, Hg, Cr and ΣPAHs). Only mercury contamination in the local harbour (S1) exceeds the regulatory N2 level. The chemical contamination increases according to (ΣQ_N1_), the sum of the ratios between contaminant concentration and N1 level in investigated sediments, as follows: S5 < S4 < S3 < S2 < S1. In addition, the global risk related to the sediment samples was investigated using the Q_PECm_, a quotient that predicts the toxic effect of contaminants in the whole sediment. Sediment samples (S1 and S2) have Q_PECm_ values greater than one, indicating that both samples may be potentially toxic, while other samples (S3, S4 and S5) have Q_PECm_ values less than one.

Therefore, prior to planned anthropogenic interventions in the local marine environment, such as the deepening of the Rovinj harbour, the results of the physico-chemical analyses of the sediment composite sample (S1) and its evaluation against the N1 and N2 pollutant values specified in the French legislation on dredging of marine and estuarine sediments (JOFR No. 184, 10-09-2000) provide information on the fate of the potential dredged material. Port sediment dredging and relocation activities are not an option because N1 contaminant limits are exceeded. Contaminants (ΣPAHs, Cu, Hg) in the S1 sediment sample exceeded the N1 level, requiring more extensive sampling, analysis and testing of the dredged material for ecotoxicity. In addition, Hg also exceeded the N2 criteria, so S1 relocation should only be conducted if the environmental impact is the lowest and requires a thorough analysis. The French SQG criteria are a binding legal act. In order to avoid time delays and reduce costs for chemical and ecotoxicological sediment analyses and necessary contaminant remediation (Hg), attention must be paid to the number of samples, which must be representative of the entire area, and sampling method (composite samples, subsamples etc.).

### 3.6. Phytotoxicity

**Figure 2:**
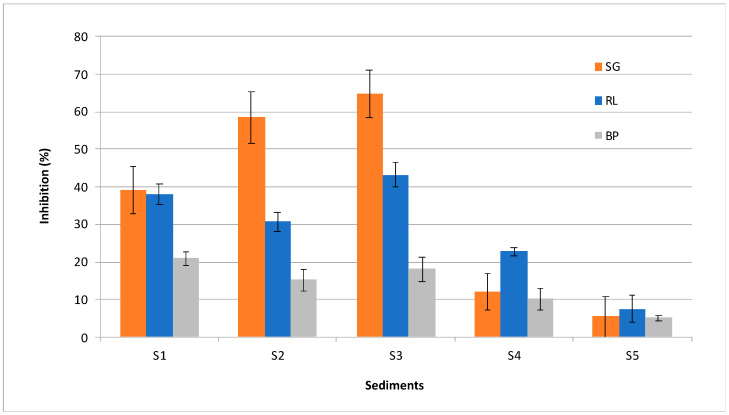
Sediment phytotoxicity shown as inhibition (%) of: germination (SG), root length (RL) and biomass root production (BP) in relation to the test negative control (0%).

The phytotoxicity assay conducted with seeds of flax *Linum usitatissimum* showed inhibition of germination, root length and root biomass production with 5 mL of sediment eluates (equivalent to 100 g/L sediment) with increasing effect: S5 < S4 < S1 < S2 < S3 (Figure 2, Table S3). In general, all three endpoints showed similar results and allowed the calculation of a phytotoxicity index (PI) (Table 2 and Table S2) using the test control (deH_2_O) as the reference value for inhibition (0%) [1]. Finally, PI allows the discrimination of two groups: S5 (6.06%) and S4 (14.99%) as less affected sediments compared to the phytotoxic sediments S1 (32.76%), S2 (34.87%) and S3 (42.00%).

Although total concentrations of contaminants in sediments can be used for ecological risk assessment, bioassays appear to be necessary to evaluate potential ecological impacts. Plant toxicity assays are also particularly important when phytotoxic contaminants are present in sediments. Seed germination and root elongation studies are often used as phytotoxicity endpoints. They have been shown to decrease significantly in soils and sediments contaminated with metals, certain pesticides and certain biocides [92]. Correlation of all phytotoxicity endpoints with other sediment contamination and risk indicators was established (Table S2). Toxicity results are not always clearly correlated with chemical concentrations in total sediments. Bioassays do not respond in the same manner as sediment chemistry analyses, suggesting that contaminant extraction/bioavailability may play an important role in observed toxicity. In phytotoxicity and marine ecotoxicology analyses, the primary focus should be on marine species and test organisms, but depending on biological effects, significance, test sensitivity, availability of organisms, experts, and equipment, including costs and benefits, statements can be made regarding the choice of the organism used: e.g., Microtox acute toxicity and SOS/*umu* tests for genotoxicity (bacteria), induction of OMF EROD (cell lines) and estrogen-androgen screening test for endocrine disruptors (yeast cells).

### 3.7. Probability of Toxic Effects

Probability of a toxic effect distinguished the studied sites according to the degree of contamination, as determined by using chemical analyses (Table 2). Sites were ranged according to increasing effect: S5 < S4 < S3 < S2 < S1. In general, sediment chemical analyses and eluate toxicity indicate studied sites S5 and S4 to be pristine areas. In addition, significant (* *p* < 0.05) correlations of P_avg_ and P_max_ values with other determined parameters were found: ΣPAHs (0.96 **; 0.81); ΣQ_N1_ (0.94 *; 0.87); Q_PECm_ (0.94 *; 0.87); SG (0.83; 0.82); RL (0.83; 0.93 *); BP (0.93 *; 0.94 *); and PI (0.88 *; 0.91 *) (Table S2). Interestingly, all measured endpoints of the phytotoxicity assay (SG, RL, BP and PI) showed significant and/or high correlation with P_avg_ and P_max_ values as indicators of potential toxicity.

### 3.8. National Sediment Quality Guidelines

The development of marine quality standards for marinas and harbours is of particular importance. This problem has already been recognized at the national level [93,94]. The collection of all data on the chemical composition of marine sediments to provide a baseline for the natural background and the development of intervention limits (thresholds) is still needed. This includes reaching consensus on which parameters, analyses, and tests should be considered and further monitored. The present study, which was only partially carried out as part of the areal, temporal and methodological time investigations, provides some data to start the initiative for Croatian national guidelines on sediment quality.

Croatian classification of pollutants in sediments can be based on the distribution of pollutant concentrations (inorganic and organic compounds and groups of compounds), harmonized similarly to the French guidelines for sediment quality: two levels (N1 and N2) and three classes: (I) below the lower value; (II) between the lower and upper value; and (III) above the upper value, as regulated by the French water policy [95]. According to this decree, the sediment is considered uncontaminated if the pollutant concentration is below the threshold N1. If the pollutant concentrations are between the N1 and N2 thresholds, the sediment is classified as contaminated, and the associated ecological impacts must be evaluated. If at least one contaminant is above the N2 threshold, the sediment is considered highly contaminated with potential ecological impact on the aquatic environment. The N1 and N2 thresholds can be derived mainly from statistical processing of physical and chemical data. In addition, for assessing the potential ecological impact of contaminated sediment classes II and III, the authors propose a simple determination of acute/chronic toxicity using standard tests (microtox, algaltox and phytotox), loosely linked to EU risk assessment principles and taking into account scientific value, availability, price and cost effectiveness.

In Europe, regulation of contaminated sediments is less coherent, with individual member states developing sediment quality guidelines (SQGs) and monitoring strategies largely independently [23]. However, sediment quality assessment is still subject to a number of uncertainties and insufficient information in terms of regulation, analytical methods, risk assessment and risk management. In general, international regulations have been translated into a national guideline directive for coastal dredged material management and marine sediment quality [96]. Contamination of marine sediments leading to toxic effects is a problem worldwide, especially in countries with a long industrial history [97]. In Croatia, industry has declined since independence in 1991, while maritime transport, tourism and aquaculture have increased [98].

The Water Framework Directive (WFD 2000/60/EC) [99] provides legislation and opportunities for monitoring and regulating the aquatic environment (MSFD 2008/56/EC, London Protocol, OSPAR Convention and Helsinki Convention) [100,101,102,103,104,105,106]. Sediments are an essential, integral, and dynamic component of the aquatic ecosystem. Healthy environments need sediments to support life, while also serving as a sink for many hazardous chemicals. Above a certain level of pollution, this leads to negative impacts such as loss of biodiversity. Although there is a link between sediment quality and the achievement of good ecological status of European waters, the WFD does not specifically address this [107]. Usually, the strategy for elaborating the guidance values is based on a statistical evaluation of the pollutant concentrations, measured during the multi-annual campaigns. The study of the distribution of the results allows determining the value of “background noise”, i.e., the natural content without any recognizable anthropological contribution. Direct comparison of national action levels/standard guidance values for sediment contaminants between countries is not possible due to the use of different classification systems, grain size fractions (standardization) in which analyses must be performed, chemical parameters (metals, organic contaminants, nutrients, etc.), and ecotoxicity bioassays where applicable. National regulations and standards for metals and organic contaminants refer, for example, to the grain size fraction: <20 μm in Germany, <63 μm in Spain and total dry weight in Denmark, Norway, Ireland, the United Kingdom, France, Belgium, the Netherlands and the Adriatic and Ionian Seas [8,9,10,26]. However, the heterogeneity of monitoring and analysis protocols may limit the comparability of data, although environmental assessment and large geographic extent require consistency. Keeping adequate documentation of monitoring and analysis protocols is essential to improve data comparability.

Recently, a document was produced within the framework of the Interreg ADRION “HarmoNIA methodological proposals” on the Adriatic-Ionian marine sub-regions, by assessment of contamination from hazardous substances (Chemical status of the WFD; Descriptor 8 of the MSFD; Ecological Objective 09 of EcAp/IMAP) and analyses of heterogeneity of monitoring procedure for the Mediterranean Region and Adriatic Sea. The greatest homogeneity between institutions was found in terms of analytical tools and sediment sampling (box corer etc.), but the thickness of the sampled sediment layer varied (surface 10, 5 and 2 cm) as well as the analyses of the different grain size fractions (<63 µm, <0.5 mm, <2 mm, unsieved) [8].

Consistent with observed gaps in regional/national harmonisation of SQGs and contaminants threshold values, the authors have undertaken a renewed campaign for sediment sampling in 2022, focusing on sediment ecotoxicological assessment, including emerging contaminants (e.g., microplastics) and determination of temporal trends, in addition to sediment physico-chemical analyses to be augmented by organic matter, carbonate content and mineral composition analyses.

## 4. Conclusions

Sediment quality guidelines (SQGs) are an important tool for assessing pollution of marine and estuarine sediments. Although such guidelines are not definitive indicators of toxicity (if toxicity tests are not included), they can have highly predictive ability and are a useful tool for identifying areas of potential adverse biological impacts.

The results and analyses represent the first evaluation of polluted coastal areas of the Adriatic Sea using French regulations based on two thresholds (N1 and N2 level) and three sediment classes.

Chemical analyses and sediment quality assessment, including phytotoxicity testing of eluates, identify sites S5 (open sea) and S4 (mariculture) as pristine.

By applying appropriate French guidelines for sediment quality (ΣQ_N1_) and probability of toxic effect (P_max_/P_avg_) it was possible to classify the studied sites according to increasing degree of pollution (S5 < S4 < S3 < S2 < S1). In addition, the general risk analysis (Q_PECm_ > 1; sites S1 and S2) can be used as an indicator of potential toxicity of sediments as an appropriate indicator of potential ecological impacts to the marine environment under study.

A similar but not identical classification was made according to phytotoxicity PI (S5 < S4 < S1 < S2 < S3). In general, bioassays do not respond in the same manner as sediment chemistry analyses, suggesting that extraction/bioavailability and the combined effects of contaminants may play a significant role in the observed “true” toxicity effects.

According to the French guidelines for the quality of marine sediments, sediments in the Rovinj area do not contain pollutants above the N2 threshold, with the exception of site S1—local harbour (mercury content). In conclusion, there is no evidence of possible negative ecological impact of human activities on the studied marine environment in the coastal area of Rovinj.

The results of our study indicate that detailed chemical and ecotoxicological analyses are needed for sediment quality evaluation of the local harbour (S1) contaminated sediments in the framework of possible further management and disposal. Unfortunately, instead of reconstruction (deepening) of the Rovinj harbour, the decision was made to extend it to a new close location in Valdibora Bay in 2020.

In order to monitor the marine environment with a special attention to marine sediments and to obtain GES, there is an urgent need to collect data on sediment quality along the eastern Adriatic in terms of pollutant concentrations, including emerging pollutants. In parallel, sediment quality standards should provide indicators of acceptability with clearly defined limits. If this legislative gap is neglected and pollution prevention procedures are not put in the place, the Eastern Adriatic will face an increase in health risks, pollution damage, a decrease in tourism activities and eventually a loss of biodiversity, in addition to not meeting the necessary environmental requirements.

## Figures and Tables

**Table 1:** Concentrations of PAHs and PAH ratios in sediments of investigated area with effect range low (ERL) and effect range median (ERM) values as a measure of toxicity.

PAHs		Sampling Sites					ERL	ERM
(µg/kg d.w.)	(Rings)	S1	S2	S3	S4	S5		
Fluorene	3	121.0 *	73.5 *	205.8 *	0.2	0.7	19.0	540
Phenanthrene (Phe)	3	976.9 *	808.3 *	773.3 *	3.0	15.3	240	1500
Anthracene (Ant)	3	196.7 *	162.7 *	156.7 *	0.4	3.2	85	1100
Fluoranthene (Flt)	4	3015.7 *	2444.5 *	1080.8 *	6.7	23.7	600	5100
Pyrene (Pyr)	4	1135.5 *	1071.7 *	668.5 *	6.1	19.6	665	2600
Benzo(a)anthracene	4	774.1 *	729.7 *	366.5 *	1.3	4.4	261	1600
Chrysene	4	707.6 *	633.9 *	290.0	2.6	12.7	384	2800
Benzo(b)fluoranthene	5	1088.7 *	1076.2 *	406.3 *	3.1	6.6	320	1880
Benzo(k)fluoranthene	5	327.5 *	331.0 *	114.9	1.2	3.2	280	1620
Benzo(a)pyrene	5	874.1 *	865.3 *	278.4	2.0	6.9	430	1600
Dibenzo(a,h)anthracene	5	113.3 *	121.1 *	25.5	0.3	0.9	63	260
Benzo(g,h,i)perylene	6	516.3 *	673.6 *	110.3 *	0.9	3.2	85	1600
Indeno(1,2,3-c,d)pyrene	6	761.9 *	875.6 *	78.9	0.5	2.4	240	950
∑PAHs		10,609.2 *	9867.1 *	4555.9 *	28.2	103.0	3672	23,150
Ant/(Ant+Phe)	3	0.17	0.17	0.17	0.12	0.17	-	-
Flt/(Flt + Pyr)	4	0.73	0.70	0.62	0.52	0.55	-	-
Phe/Ant	3	4.97	4.97	4.93	7.04	4.74	-	-
Flt/Pyr	4	2.66	2.28	1.62	1.10	1.21	-	-

* Values above ERL level.

**Table 2:** Results of sediment chemical analyses and contamination evaluation: ΣQ_N1_—cumulative risk quotients, Q_PECm_—averaged risk quotient according to French N1 and N2 level regulatory management, (P_avg_, P_max_)—probability of toxic effects and (PI)—Phytotoxicity index.

Parameters (Units)	Sampling Sites					N1	N2
	S1 Harbour	S2 Shipyard	S3 Lim Out	S4 Lim Middle	S5 Open Sea	Legal Level	Legal Level
As/(mg/kg d.w.)	9.223	23.440	8.126	13.850	3.985	25	50
Cd/(mg/kg d.w.)	0.265	0.092	0.083	0.091	0.073	1.2	2.4
Cu/(mg/kg d.w.)	69.95 *	30.590	13.770	18.450	4.770	45	90
Ni/(mg/kg d.w.)	7.930	14.150	28.59	41.160 *	8.490	37	74
Pb/(mg/kg d.w.)	24.380	6.950	1.830	1.350	3.690	100	200
Zn/(mg/kg d.w.)	115.60	50.66	71.75	88.000	31.870	276	552
Hg/(mg/kg d.w.)	0.838 *^,^**	0.266	0.129	0.138	0.038	0.4	0.8
Cr/(mg/kg d.w.)	22.490	26.31	76.11	98.14 *	22.390	90	180
ΣPAHs/(mg/kg d.w.)	10.609 *	9.867 *	4.555 *	0.028	0.103	1.5	15
ΣPCBs/(mg/kg d.w.)	0.278	0.170	0.058	0.021	<0.010	0.5	1.0
ΣQ_N1_	12.99	10.20	6.07	3.98	1.12	-	-
Q_PECm_	1.30	1.02	0.61	0.40	0.11	-	-
P_avg_	0.81	0.73	0.62	0.24	0.18	-	-
P_max_	0.51	0.48	0.47	0.35	0.15	-	-
**Phytotoxicity (PI%)**	32.76	34.87	42.00	14.99	6.06	-	-

* Values above N1, and ** values above N2 level.

## Data Availability

The data presented in this study are available on request from the corresponding author.

## Supplementary Materials

The following supporting information can be downloaded at: https://www.mdpi.com/article/10.3390/toxics10080478/s1, Table S1: Grain size fractions of marine sediments; Table S2: Correlation coefficients (r) of metals (As, Cd, Cu, Ni, Pb, Zn, Hg, Cr), total ΣPAHs and ΣPCBs, ∑QN1 and QPECm evaluation, probabilities of a toxic effect (Pavg and Pmax), and Phytotoxicity (SG, RL, BP, PI) results of marine sediments contamination analyses; Table S3: Phytotoxicity of investigates marine sediment eluates using dicotyledon flax Linum usitatissimum seed germination test: (A) seed germination (GP), (B) root length (RL), (C) root biomass production (BP) inhibition and phytotoxicity index (PI) calculated using test control (deH2O) as 0.00% inhibition; Figure S1 Polyclic Aromatic Hydrocarbons structure distribution patterns in Rovinj marine sediments.
