# Supplementary material for: Physico-Chemical and Ecotoxicological Evaluation of Marine Sediments Contamination: A Case Study of Rovinj Coastal Area, NE Adriatic Sea, Croatia
Source: Toxics. 2022 Aug 16;10(8):478. doi: 10.3390/toxics10080478 (PMC9415096; doi:10.3390/toxics10080478)
Supplement: Supplementary file 1 [file toxics-10-00478-s001.zip › toxics-1808778-supplementary.pdf]

toxics

Table S1. Grain size data of marine sediments.

Location		S1 Harbour	S2 Shipyard	S3 Lim Bay out	S4 Lim Bay middle	S5 Open Sea
**Total gravel**	**> 2 mm (%)**	13.04	0.74	0.27	0.41	11.07
**Total sand**	**63 µm – 2 mm (%)**	67.32	54.17	5.44	4.55	72.60
**Total mud**	**< 63 µm (%)**	19.64	45.09	94.29	95.04	16.33
**Sediment type**		slightly gravelly muddy **sand**	gravelly muddy **sand**	slightly gravelly muddy **sand**	sandy gravelly **mud**	slightly gravelly **mud**
**Mean size (µm)**		167	72	45	45	175
**Sorting**		very poorly	poorly	well	very well	very poorly

toxics

Table S2. Correlation coefficients (r) of metals (As, Cd, Cu, Ni, Pb, Zn, Hg, Cr), total ΣPAHs and ΣPCBs, $\sum Q_{N1}$ and Q_{PECm} evaluation, probabilities of a toxic effect (P_{avg} and P_{max}), and Phytotoxicity (SG, RL, BP, PI) results of marine sediments contamination analyses.

	As	Cd	Cu	Ni	Pb	Zn	Hg	Cr	∑PAHs	∑PCBs	P_{avg}	P_{max}	$\sum Q_{N1}$	Q_{PECm}	SG	RL	BP	PI
**As**	-	-0.11	0.18	0.15	-0.04	-0.01	0.06	0.01	0.47	0.31	0.40	0.51	0.46	0.46	0.43	0.24	0.23	0.36
**Cd**	-0.11	-	0.96*	0.42	0.97**	0.79	0.98**	-0.38	0.64	0.86	0.61	0.51	0.76	0.76	0.10	0.43	0.66	0.29
**Cu**	0.18	0.96*	-	-0.39	0.95*	0.77	0.99**	-0.39	0.80	0.95*	0.74	0.67	0.90*	0.90*	0.26	0.52	0.75	0.42
**Ni**	0.15	0.42	-0.39	-	-0.60	0.21	-0.44	0.99**	-0.51	-0.54	-0.35	0.07	-0.35	-0.34	-0.05	0.14	-0.10	0.00
**Pb**	-0.04	0.97**	0.95*	-0.60	-	0.064	0.98**	-0.58	0.73	0.92*	0.65	0.47	0.80	0.80	0.14	0.36	0.63	0.28
**Zn**	-0.01	0.79	0.77	0.21	0.064	-	0.77	0.25	0.39	0.58	0.46	0.64	0.62	0.62	0.14	0.61	0.70	0.37
**Hg**	0.06	0.98**	0.99**	-0.44	0.98**	0.77	-	-0.42	0.77	0.94*	0.72	0.62	0.86	0.86	0.23	0.50	0.73	0.40
**Cr**	0.01	-0.38	-0.39	0.99**	-0.58	0.25	-0.42	-	-0.53	-0.55	-0.35	0.06	-0.37	-0.36	-0.05	0.18	-0.06	0.02
**∑PAHs**	0.47	0.64	0.80	-0.51	0.73	0.39	0.77	-0.53	-	0.94*	0.96**	0.81	0.96**	0.96**	0.70	0.66	0.82	0.74
**∑PCBs**	0.31	0.86	0.95*	-0.54	0.92*	0.58	0.94*	-0.55	0.94*	-	0.87	0.72	0.96**	0.96**	0.46	0.56	0.78	0.56
**P_{avg}**	0.40	0.61	0.74	-0.35	0.65	0.46	0.72	-0.35	0.96**	0.87	-	0.90*	0.94*	0.94*	0.83	0.83	0.93*	0.88*
**P_{max}**	0.51	0.51	0.67	0.07	0.47	0.64	0.62	0.06	0.81	0.72	0.90	-	0.87	0.87	0.82	0.93*	0.94*	0.91*
**$\sum Q_{N1}$**	0.46	0.76	0.90*	-0.35	0.80	0.62	0.86	-0.37	0.96**	0.96**	0.94	0.87	-	1**	0.62	0.72	0.88	0.72
**Q_{PECm}**	0.46	0.76	0.90*	-0.34	0.80	0.62	0.86	-0.36	0.96**	0.96**	0.94	0.87	1**	-	0.62	0.72	0.88*	0.72
**SG**	0.43	0.10	0.26	-0.05	0.14	0.14	0.23	-0.05	0.70	0.46	0.83	0.82*	0.62	0.62	-	0.86	0.78	0.97**

**RL**	0.24	0.43	0.52	0.14	0.36	0.61	0.50	0.18	0.66	0.56	0.83	0.93*	0.72	0.72	0.86	-	0.95*	0.95*
**BP**	0.23	0.66	0.75	-0.10	0.63	0.70	0.73	-0.06	0.82	0.78	0.93*	0.94*	0.88	0.88*	0.78	0.95*	-	0.90*
**PI**	0.36	0.29	0.42	0.00	0.28	0.37	0.40	0.02	0.74	0.56	0.88*	0.91*	0.72	0.72	0.97**	0.95*	0.90*	-

Table S3. Phytotoxicity of investigates marine sediment eluates using dicotyledon flax *Linum usitatissimum* seed germination test: A) seed germination (GP), B) root length (RL), C) root biomass production (BP) inhibition and phytotoxicity index (PI) calculated using test control (deH2O) as 0.00 % inhibition.

A) Seed Germination Inhibition - SG

30 x 30 seeds of *L. usitatissimum*

Plate: A Subsample	Seed Germination (SG)	Sample Unit	Control	S1	S2	S3	S4	S5
1		(No)	27	15	9	11	24	26
2		(No)	26	12	10	13	25	26
3		(No)	27	16	9	9	25	27
	Germinated seeds	(Mean)	26.67	14.33	9.33	11.00	24.67	26.33
	Germination	(%)	88.89	47.78	31.11	36.67	82.22	87.78
	Seed Germination	(%)	0.00	46.25	65.00	58.75	7.50	1.25

30 x 30 seeds of *L. usitatissimum*

Plate: B Subsample	Seed Germination (SG)	Sample Unit	Control	S1	S2	S3	S4	S5
1		(No)	25	16	12	8	23	27
2		(No)	28	18	13	6	23	25
3		(No)	27	18	14	9	25	25
	Germinated seeds	(Mean)	26.67	17.33	13.00	7.67	23.67	25.67
	Germination	(%)	88.89	57.78	43.33	25.56	78.89	85.56
	Seed Germination	(%)	0.00	35.00	51.25	71.25	11.25	3.75

30 x 30 seeds of *L. usitatissimum*

Plate: C Subsample	Seed Germination (SG)	Sample Unit	Control	S1	S2	S3	S4	S5
1		(No)	29	17	10	9	25	25
2		(No)	30	18	11	10	23	25
3		(No)	27	20	14	12	23	26
	Germinated seeds	(Mean)	28.67	18.33	11.67	10.33	23.67	25.33
	Germination	(%)	95.56	61.11	38.89	34.44	78.89	84.44
	Seed Germination	(%)	0.00	36.05	59.30	63.95	17.44	11.63

A, B, C	SG	Average	0.00	39.10	58.52	64.65*	12.06	5.54*
		StDev	0.00	6.22	6.91	6.28	5.02	5.42

Kruskall-Wallis ANOVA P = 0.014; S3-S5 P = 0.047

B) Root Lenght Inhibition - RL

30 x 30 seeds of *L. usitatissimum*

Plate: A Subsample	Root lenght (RL)	Sample Unit	Control	S1	S2	S3	S4	S5
1	Root Lenght	(mm)	85.9	50.3	59.3	48.2	67.7	81
	RL	(%)	0.00	41.44	30.97	43.89	21.19	5.70
2	Root Lenght	(g)	78.7	53.2	58.9	50.2	64.8	74.4
	RL	(%)	0.00	32.40	25.16	36.21	17.66	5.46
3	Root Lenght	(g)	83.5	49.4	51.4	45.2	58.6	80.3
	RL	(%)	0.00	40.84	38.44	45.87	29.82	3.83

RL	Average	0.00	38.23	31.52	41.99	22.89	5.00
	StDev	0.00	5.05	6.66	5.10	6.26	1.02

Plate: B Subsample	Root lenght (RL)	Sample Unit	Control	S1	S2	S3	S4	S5
1	Root Lenght	(mm)	81.6	52.1	61.4	45.3	63.4	78.3
	RL	(%)	0.00	36.15	24.75	44.49	22.30	4.04
2	Root Lenght	(g)	78.2	58.6	64.7	51.3	66.5	72.2
	RL	(%)	0.00	25.06	17.26	34.40	14.96	7.67
3	Root Lenght	(g)	85.2	46.7	49.3	47.9	61.7	79.8
	RL	(%)	0.00	45.19	42.14	43.78	27.58	6.34

RL	Average	0.00	35.47	28.05	40.89	21.62	6.02
	StDev	0.00	10.08	12.76	5.63	6.34	1.84

Plate: C Subsample	Root lenght (RL)	Sample Unit	Control	S1	S2	S3	S4	S5
1	Root Lenght	(mm)	91.4	53.7	56.9	44.1	70.1	73.5
	RL	(%)	0.00	41.25	37.75	51.75	23.30	19.58
2	Root Lenght	(g)	78.7	55.4	58.5	47.5	61.5	74.1
	RL	(%)	0.00	29.61	25.67	39.64	21.86	5.84
3	Root Lenght	(g)	88.2	40.8	53.9	42.3	61.8	75.9
	RL	(%)	0.00	51.14	35.45	49.34	25.99	9.10

RL	Average	0.00	40.66	32.95	46.91	23.72	11.51
	StDev	0.00	10.78	6.41	6.41	2.10	7.18

A, B, C	RL	Average	0.00	38.12	30.84	43.26*	22.74	7.51*
		StDev	0.00	2.60	2.52	3.21	1.06	3.50

Kruskall-Wallis ANOVA P = 0.009; S3-S5 P = 0.01

C) Root Biomass Inhibition - BP

30 x 30 seeds of *L. usitatissimum*

Plate: A Subsample	Biomass production (BP)	Sample Unit	Control	S1	S2	S3	S4	S5
1	Root biomass	(g)	0.253	0.195	0.205	0.197	0.227	0.243
	BP	(%)	0.00	22.92	18.97	22.13	10.28	3.95
2	Root biomass	(g)	0.238	0.189	0.206	0.193	0.218	0.221
	BP	(%)	0.00	20.59	13.45	1.91	8.40	7.14
3	Root biomass	(g)	0.285	0.211	0.242	0.235	0.265	0.271
	BP	(%)	0.00	25.96	15.09	17.54	7.02	4.91

BP	Average	0.00	23.16	15.84	19.53	8.57	5.34
	StDev	0.00	2.70	2.84	2.36	1.64	1.64

Plate: B Subsample	Biomass production (BP)	Sample Unit	Control	S1	S2	S3	S4	S5
1	Root biomass	(g)	0.274	0.201	0.218	0.193	0.235	0.254
	BP	(%)	0.00	26.64	20.44	29.56	14.23	7.30
2	Root biomass	(g)	0.246	0.193	0.196	0.201	0.211	0.231
	BP	(%)	0.00	21.54	20.33	18.29	14.23	6.10
3	Root biomass	(g)	0.254	0.223	0.222	0.221	0.224	0.245
	BP	(%)	0.00	12.20	12.60	12.99	11.81	3.54

BP	Average	0.00	20.13	17.79	20.28	13.42	5.65
	StDev	0.00	7.32	4.49	8.46	1.40	1.92

Plate: C Subsample	Biomass production (BP)	Sample Unit	Control	S1	S2	S3	S4	S5
1	Root biomass	(g)	0.266	0.233	0.241	0.248	0.24	0.258
	BP	(%)	0.00	13.04	9.88	7.11	10.28	3.16
2	Root biomass	(g)	0.245	0.195	0.217	0.201	0.228	0.233
	BP	(%)	0.00	20.41	11.43	17.96	6.94	4.90
3	Root biomass	(g)	0.274	0.202	0.233	0.224	0.251	0.26
	BP	(%)	0.00	26.28	14.96	18.25	8.39	5.11

BP	Average	0.00	19.91	12.09	14.44	8.54	4.39
	StDev	0.00	6.63	2.61	6.35	1.67	1.07

A, B, C	BP	Average	0.00	21.01*	15.24	18.08	10.18	5.12*
		StDev	0.00	1.82	2.89	3.18	2.81	0.65

Kruskall-Wallis ANOVA P = 0.017; S1-S5 P = 0.019

Germination test results	Sample Unit	Control	S1	S2	S3	S4	S5
SG	(%)	0	39.10	58.52	64.65	12.06	5.54
RL	(%)	0	38.12	30.84	43.26	22.74	7.51
BP	(%)	0	21.07	15.24	18.08	10.18	5.12
PHYTOTOXICITY INDEX (PI)	AVERAGE	0	32.76	34.87	42.00	14.99	6.06
	StDev	0	10.14	21.92	23.31	6.78	1.27

toxics

MDPI

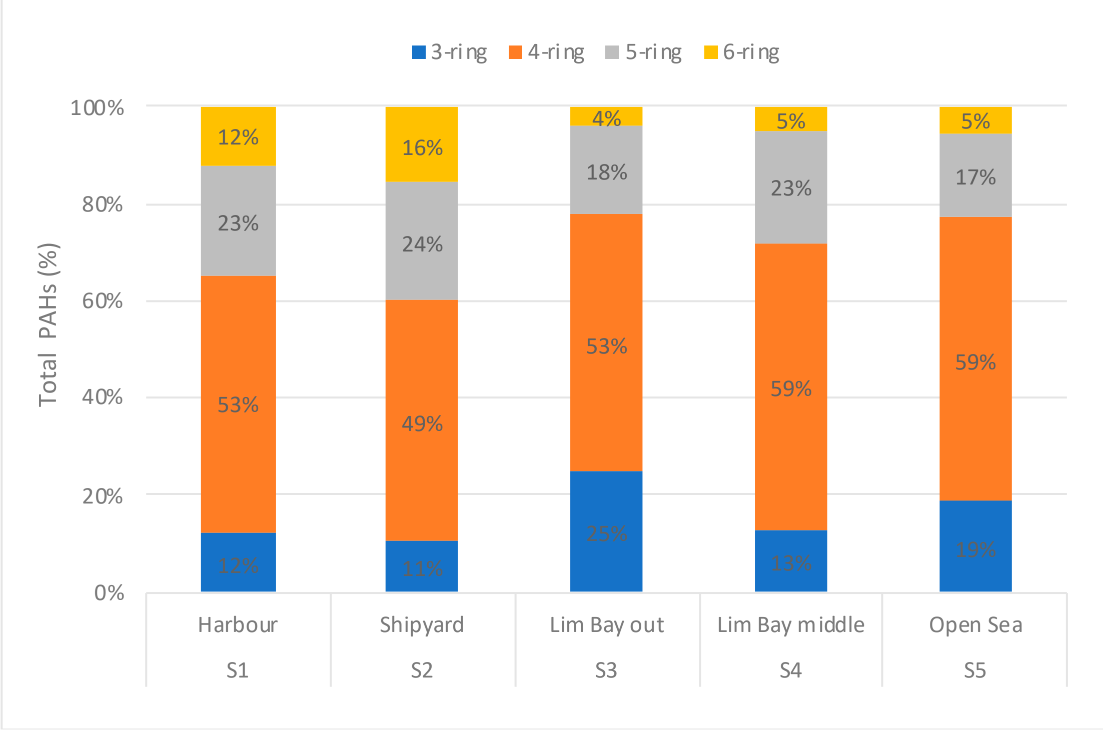

Figure S1. Polycyclic Aromatic Hydrocarbons structure distribution patterns in Rovinj marine sediments.
